# Intervention reporting by the Landesgesundheitskonferenz Berlin (Berlin Health Conference)

**DOI:** 10.17886/RKI-GBE-2017-096

**Published:** 2017-08-30

**Authors:** Susanne Bettge, Sylke Oberwöhrmann

**Affiliations:** Berlin Senate Administration for Health, Care and Equality, Department for Health Reporting, Epidemiology, Health Information Systems, Statistics Office

## Abstract

In 2013 the Berlin Health Conference (Landesgesundheitskonferenz, LGK) implemented intervention reporting for the first time within a kindergarten setting. Results from the survey of LGK Berlin members on current measures in kindergartens in Berlin that are related to the child health targets enabled us to map socio-spatial interventions and subsequently identify needs. This article highlights the potential and limits of intervention reporting as an element in the wider field of health and prevention reporting.

## Background

The LGK Berlin is a network of institutions and associations that actively partake in health policy at the federal state level in Berlin and/or hold health policy responsibility. In 2012 the LGK decided to begin internal intervention reporting. Intervention reporting aims to create an instrument to optimise the management of activities and will be based on the self-commitment of LGK members to transparency in the implementation of measures in the context of Berlin's health target process [1].

## Possibilities and limits of intervention reporting

Intervention reporting was implemented for the first time in 2013 referring to measures in a kindergarten setting. LGK members were asked which measures they were implementing in kindergartens that relate to the fields of activity for Berlin's child health targets. To judge whether offers meet the (estimated) needs, the locations of the kindergartens in which LGK stakeholders were implementing measures were projected onto a map providing information on Berlin's social structure [2]. Whilst measures are clearly concentrated in socially disadvantaged quarters of the city, there are still disadvantaged areas in which no measures are being implemented, and also kindergartens in more socially favourable areas where measures are nonetheless being implemented.

Most LGK stakeholders view intervention reporting as a useful tool to identify social spaces with a heightened need for measures and those where needs are already being satisfied. Reporting can help account for socially compensatory aspects in the planning of measures. One risk is the potential to misinterpret results: a high number of offers in a particular area does not automatically mean that health is being effectively promoted here and vice versa, particularly because whilst intervention reporting does provide information on the kind and number of measures, it cannot say anything about how effective these measures were. Because intervention reporting only collects information on activities by the members of the LGK, it also does not provide a full picture of the activities in a social space that are directed at promoting health.

## The relation between health, intervention and prevention reporting

Generally speaking, intervention reporting is a potential element of prevention reporting. Meaningful prevention reporting should cover all phases of the Public Health Action Cycle (assessment, policy development, assurance and evaluation) [3, 4]. Health reporting can help identify where needs exist. The development of intervention strategies is a task limited to health care stakeholders and can be directed and co-ordinated by committees such as the LGK Berlin. Intervention reporting can provide findings on the implementation and complementarity of offers and needs, as well as, under certain circumstances, on the degree to which target groups have been reached. Prevention reporting, however, fundamentally also depends on information on the efficacy of measures and the means applied. Successful disease prevention and health promotion measures should in turn be reflected in the data produced by health reporting.

## References

[1] Senatsverwaltung für Gesundheit, Pflege und Gleichstellung, Abteilung Gesundheit (2017) Gesundheitsziele. www.berlin.de/sen/gesundheit/themen/gesundheitsfoerderung-und-praevention/landesgesundheitskonferenz-berlin/gesundheitsziele/ (As at 15.05.2017)

[2] Meinlschmidt G (2014) Handlungsorientierter Sozialstrukturatlas Berlin 2013. Ein Instrument der quantitativen, interregionalen und intertemporalen Sozialraumanalyse und -planung. Gesundheitsberichterstattung Berlin, Spezialbericht 2014 – 1. Senatsverwaltung für Gesundheit und Soziales, Berlin

[3] Rosenbrock R (1995) Public Health als soziale Innovation. Gesundheitswesen 57(3):140-144 7756762

[4] Ruckstuhl B Somaini B Twisselmann W (2008) Förderung der Qualität in Gesundheitsprojekten. Der Public Health Action Cycle als Arbeitsinstrument. Institut für Sozial- und Präventivmedizin, Zürich und Bundesamt für Gesundheit, Bern

